# Supplementary figures and images for: Large-scale genetic characterization of the model sulfate-reducing bacterium, Desulfovibrio vulgaris Hildenborough
Source: Front Microbiol. 2023 Mar 31;14:1095191. doi: 10.3389/fmicb.2023.1095191 (PMC10102598; doi:10.3389/fmicb.2023.1095191)

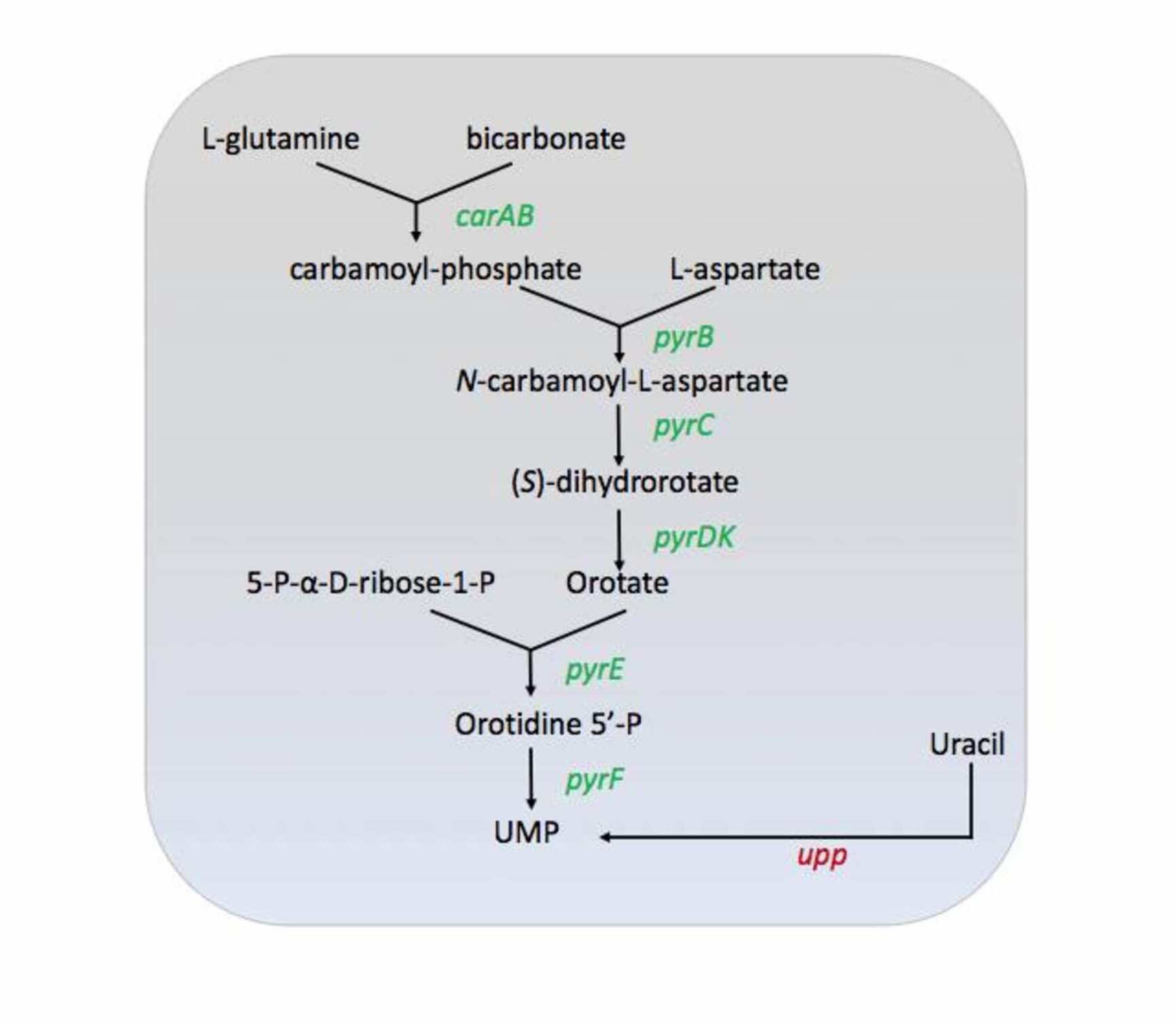

Supplement: SUPPLEMENTARY FIGURE S1 — Genes of the de novo UMP biosynthesis pathway are marked in green, all of these genes are essential for viability in the JW710 background that contains a deletion of upp. In contrast, all 8 of these genes are non-essential in the wild-type DvH background, as the intact upp provides a route to synthesize UMP. [file Image_1.JPEG]

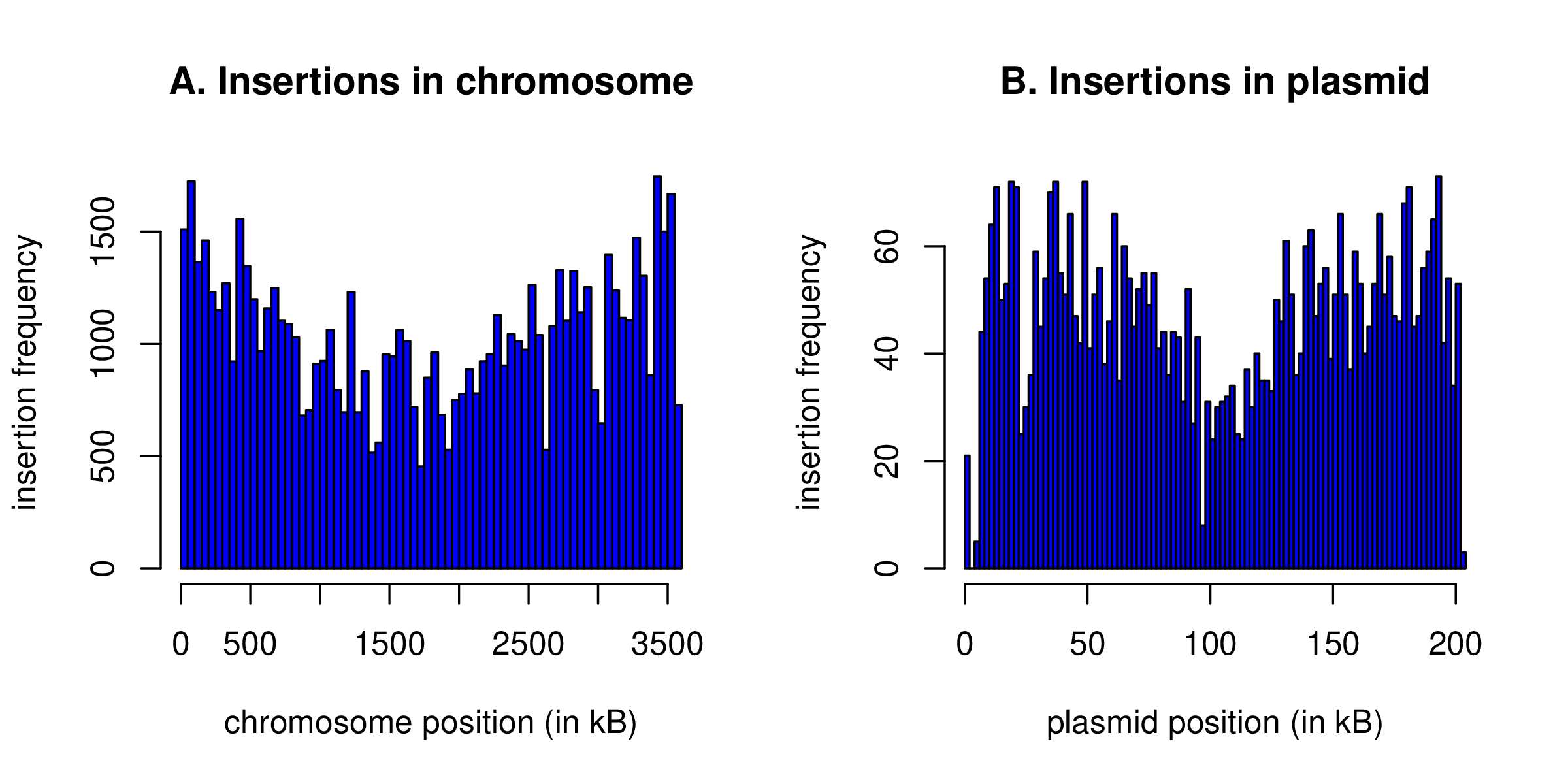

Supplement: SUPPLEMENTARY FIGURE S2 — Insertion coverage of mapped transposon insertions in the DvH JW710 RB-TnSeq mutant library across the chromosome (A) and megaplasmid (B). [file Image_2.JPEG]

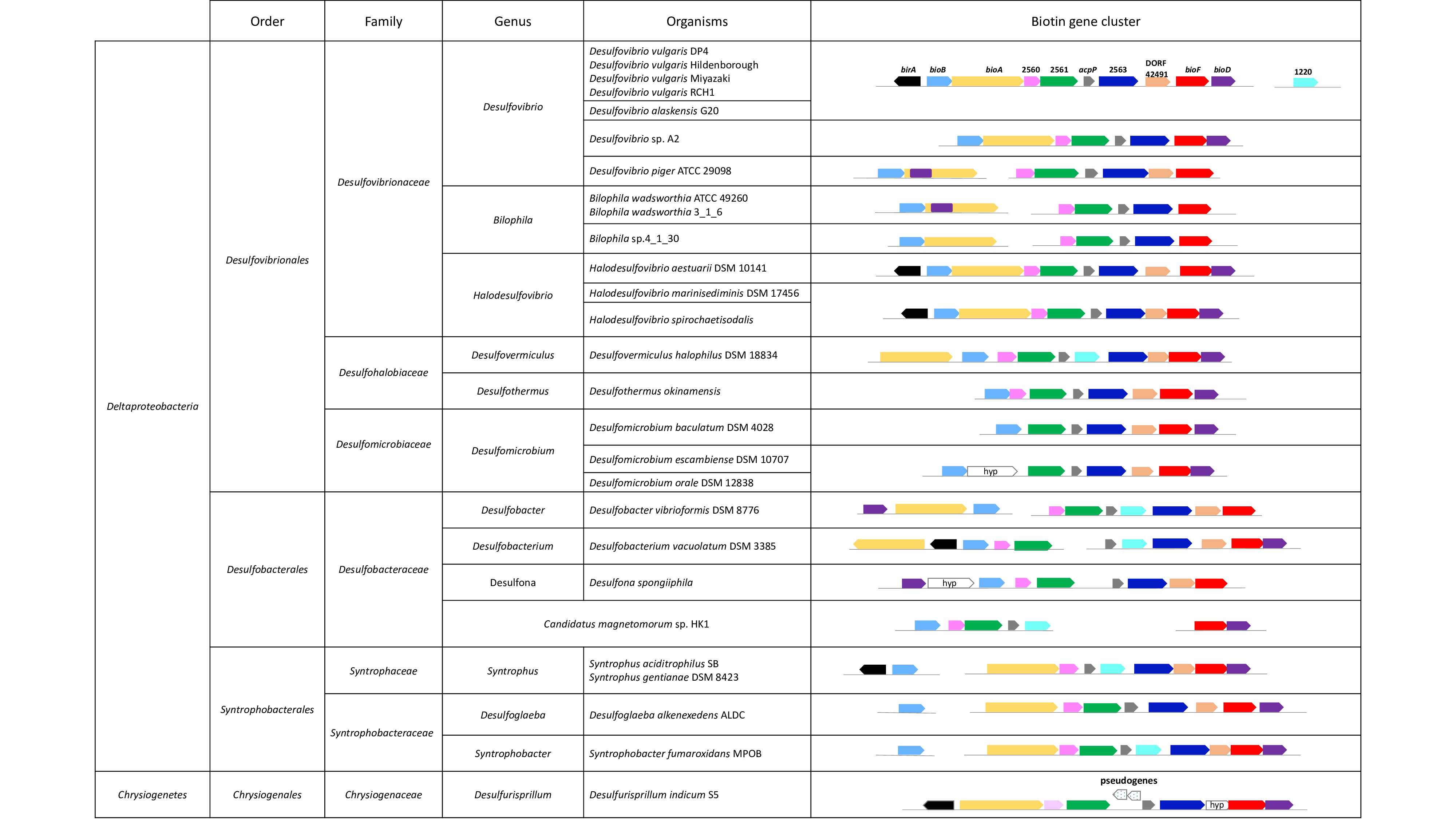

Supplement: SUPPLEMENTARY FIGURE S3 — Biotin cluster genes conservation in the Desulfobacterota and Chrysiogenetes. The birA genes are shown only when located in the direct vicinity of a bio gene. In Halodesulfovibrio spirochaetisodalis genome, the gene homolog to DvH DORF41491 was added to the original annotation. The regions of Desulfobacterium vacuolatum DSM 3385 genome shown in the figure represent the end and beginning of two separate contigs. [file Image_3.JPEG]

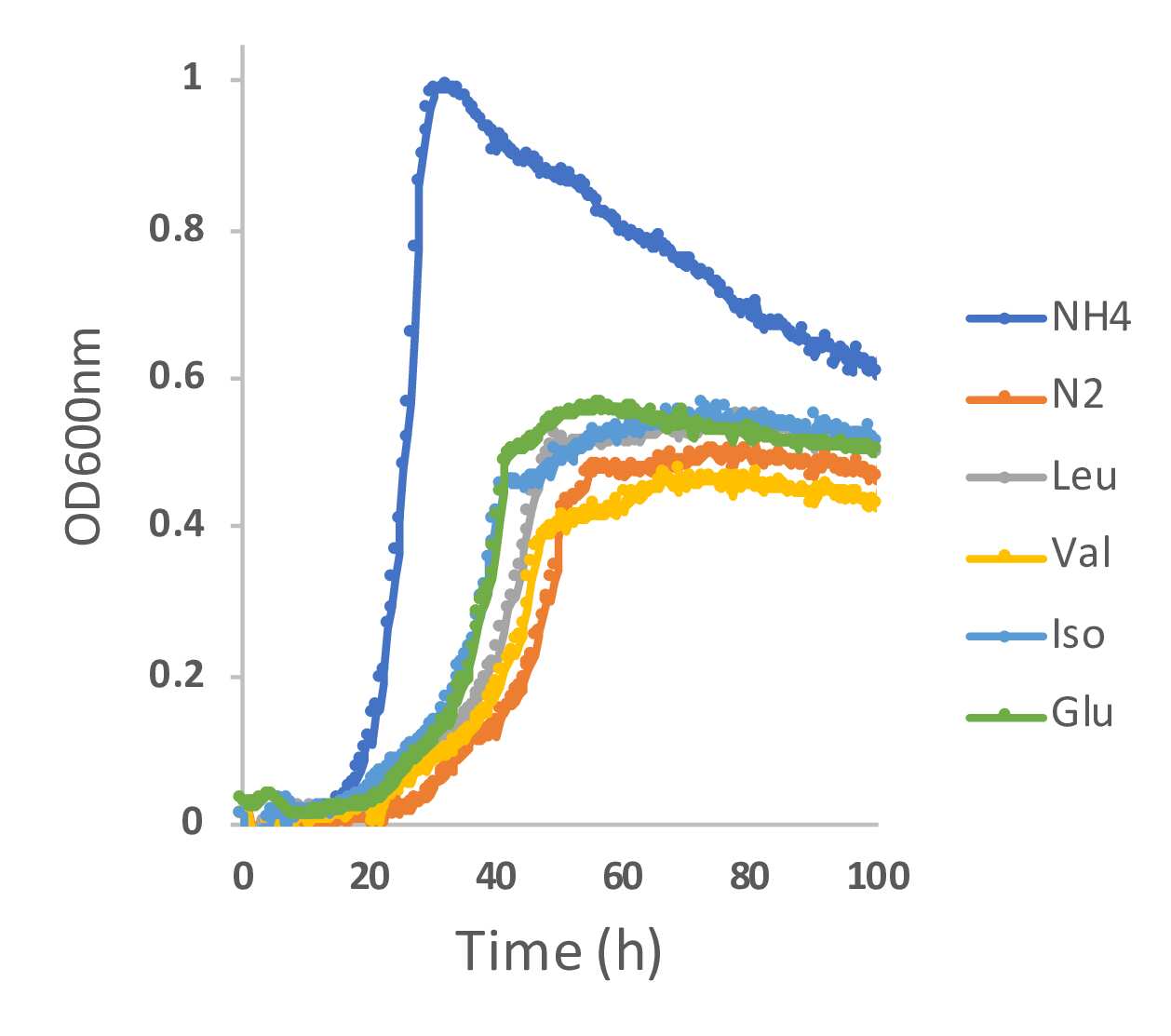

Supplement: SUPPLEMENTARY FIGURE S4 — Growth of DvH in lactate-sulfate minimal medium with different compounds as the sole source of nitrogen: N2(90%atm), NH4, valine, leucine, isoleucine and glutamate (20mM). Measurements were made in a Bioscreen growth analysis system and each curve is the average of four replicates. [file Image_4.JPEG]
